# Circular mRNA-LNP vaccine encoding self-assembled E2-TMD-mi3 nanoparticles licit enhanced CSFV-specific immunity over commercial subunit vaccine

**DOI:** 10.3389/fimmu.2025.1604677

**Published:** 2025-06-19

**Authors:** Chunxi Liu, Weifeng Zhai, Wenhao Nie, Conghao Zhong, Feifei Diao, Bo Yin

**Affiliations:** ^1^ Research and Development (R&D) Center, Shanghai ShenRay United Biomedical Co., Ltd., Shanghai, China; ^2^ Research and Development (R&D) Center, Shanghai ShenLian Biomedical Corporation, Shanghai, China; ^3^ National University of Singapore (Suzhou) Research Institute, Suzhou, China

**Keywords:** classical swine fever virus, E2 glycoprotein, mi3 self-assembled nanoparticles, AX4-LNP, cmRNA-LNP vaccine

## Abstract

The E2 subunit vaccine is crucial for eliminating Classical Swine Fever Virus (CSFV) due to its favorable biosafety and Differentiating Infected from Vaccinated Animals (DIVA) capability. However, low immunogenicity and high costs limit its broader application. To overcome these bottlenecks, we leveraged mRNA-LNP technology to design next-generation E2 glycoprotein vaccines with enhanced immunogenicity and cost-effectiveness. We designed different E2 glycoprotein coding sequences incorporating CD154 adjuvants and mi3 self-assembled nanoparticles, delivered via cmRNA-LNP formulations in murine immunogenicity testing. Among these, E2-TMD-mi3 cmRNA-LNP vaccine induced high-titer antibodies with a 78.25% ± 1.32% blocking rate at day 14 post-booster, significantly higher than the commercial subunit vaccine (39.74% ± 3.30%, *p*<0.01). To further optimize vaccine performance, we compared cmRNA-LNP formulations incorporating with different cationic lipids. Notably, AX4-LNP formulation induced superior cellular and humoral immunity compared to other cationic lipids. In mice, this vaccine induced robust humoral immunity, achieving a mean blocking rate of 80.55% ± 2.06% by day 14 post-booster, alongside potent cellular immunity (IFN-γ ELISpot, 319.60 ± 45.23 SFC/10^5^ cell, 5.6-fold higher than that of the commercial vaccine). In swine, the CSFV-specific antibody blocking rate remained at 54.76% ± 3.21% at 120 days post-primary vaccination. In contrast, the antibody blocking rates in other cmRNA-LNP vaccine groups and the commercial vaccine group were below the positivity threshold (<40%, set according to the manufacturer’s technical specifications), outperforming commercial subunit vaccines. Moreover, this vaccine does not affect the body weight gain of immunized pigs and does not cause inflammatory reactions at the immunization site. Ultimately, we successfully developed a cmRNA-LNP vaccine incorporating the E2-TMD-Mi3 coding sequence and AX4-LNP, which demonstrated superior immunogenicity compared to commercial subunit vaccines. This study establishes a modular cmRNA-LNP platform combining mi3 nanoparticles, overcoming traditional subunit vaccine limitations for porcine viral pathogens.

## 1 Introduction

Classical swine fever (CSF), characterized by its high contagiousness and mortality, has been documented in major pork-producing regions with intensive farming practices, posing severe threats to the global swine industry (1). The causative agent of CSF is classical swine fever virus (CSFV) (2), which is classified within the *Pestivirus* genus of the *Flaviviridae* family (3). CSFV is an enveloped virus harboring a single-stranded RNA genome approximately 12.3 kb in length, which consists of an open reading frame (ORF) flanked by 5’ and 3’ untranslated regions (UTRs) (4). This ORF encodes a polyprotein of 3898 amino acids which undergoes post-translational processing mediated by both viral proteases and host cellular enzymes (5–7). The cleavage generates multiple functional virus proteins, including four structural proteins (Core, E^rns^, E1, and E2) and eight non-structural proteins (N^pro^, p7, NS2, NS3, NS4A, NS4B, NS5A, and NS5B) (8).

Biosecurity measures and vaccination currently remain crucial strategies for the prevention and control of CSF (9). Owing to the extensive deployment of the attenuated live vaccines such as hog cholera lapinized virus (HCLV), the CSF epidemic in China has been well controlled (10). However, the use of attenuated live vaccines makes it difficult to differentiate between infected and vaccinated animals (DIVA) which is very important for the elimination of CSF, thereby necessitating the urgent development of novel DIVA-capable vaccine platforms (11–13).

As the major envelope glycoprotein, E2 is not only critical for the attachment and entry of the virus (14–16) but also serves as a key target for immune responses (17). E2 glycoprotein obtained from different protein expression systems have been used to develop subunit vaccines (17–20). Among these, the commercial subunit vaccine employed in this study is expressed via a recombinant baculovirus expression system, which facilitates proper glycosylation modification of the E2 glycoprotein and preserves its native biological activity. The purified inactivated E2 protein is emulsified with an oil-based adjuvant to generate a water-in-oil emulsion formulation, featuring enhanced sustained-release kinetics that potently elicit robust humoral immune responses. This vaccine not only exhibit good immunoprotective efficacy but also enable DIVA, thereby serving as a strategic tool for the elimination of CSFV (21). However, E2 glycoprotein subunit vaccines exhibit inferior immune efficacy compared to live attenuated vaccines. They require more time to induce neutralizing antibody production and fail to elicit high-level cellular immunity (13). How to enhance the immunogenicity of E2 protein has become the focus of the development of vaccines based on E2 glycoprotein. Subunit vaccines based on the E2 glycoprotein are typically formulated by emulsifying *in vitro*-expressed E2 protein with adjuvants to form oil-in-water emulsions. Thus, adjuvant selection is key to improving E2 subunit vaccine immunogenicity. Common oil-based adjuvants like Montanide ISA 15A VG and ISA 50 V2 exhibit strong immunostimulatory effects (22). Additionally, food-grade saponin extracts offer both emulsification and immunostimulatory benefits (23). Given the high cost and toxicity of traditional oil-based adjuvants and food-grade saponin extracts, and as CSFV vaccine development diversifies, strategies to improve E2 immunogenicity via fusion with self-assembling nanoparticles and immunostimulatory molecular adjuvants have become increasingly prevalent in CSFV vaccine research. Linking the immunogenic domains of viral structural proteins to the surface of self-assembling nanoparticles such as mi3 is an important strategy for enhancing the immunogenicity of viral structural proteins (24). Mi3 is a spontaneously assembled icosahedral cage-like protein nanoparticle with 60 subunits (25). Its highly ordered and repetitive structure allows it to act as a protein scaffold, boosting the antigen density of target proteins cross-linked to its surface (25). Additionally, mi3’s relatively large molecular size (typically larger than 25 nm) facilitates its uptake by antigen-presenting cells (APCs) (26). In contrast to the self-assembled nanoparticle strategy, which modifies the intrinsic properties of antigenic proteins, molecular adjuvants like CD40L(CD154) ligand (27, 28) (a member of the tumor necrosis factor superfamily) predominantly exhibit immunostimulatory properties. They bolster the host’s immune response by triggering the activation and maturation of immune cells, including dendritic cells and B cells, thus enhancing vaccine efficacy (27). Both strategies have been applied in the development of the E2 glycoprotein subunit vaccine (27, 29).

Notably, the above-mentioned E2 glycoprotein immunogenicity improvement strategies often rely on eukaryotic expression systems for vaccine production, which leads to a long R & D cycle and high production costs. Unlike traditional vaccines that depend on *in vitro* expression systems, mRNA vaccines are produced through cell-free *in vitro* transcription. This approach eliminates the requirement for live cell cultures, making the production process more suitable for large-scale manufacturing (30). Moreover, compared with mRNA, circular messenger RNA (cmRNA), which also belongs to the RNA family, has better stability because of its unique circular structure that can avoid degradation by exonucleases (31). This not only reduces the transportation and storage costs of vaccines but also prolongs the half-life of cmRNA in host cells, thereby increasing the expression level of the target protein in host cells (31). Significantly, the delivery efficiency of cmRNA is a key factor determining the immunological effect of cmRNA vaccines. Due to their excellent stability and biocompatibility, lipid-based nanoparticles (LNP) are widely used in the *in vivo* delivery of cmRNA (32). LNP-formulated cmRNA has emerged as a promising preclinical research hotspot for vaccine development and cancer treatment (33, 34). Obviously, delivering the optimized sequence of E2 glycoprotein via the cmRNA-LNP vaccine platform could overcome these limitations for developing DIVA-capable vaccines. Therefore, in this study, this study systematically evaluated 5 candidate encoding sequences in murine models, identifying the E2-TMD-Mi3 sequence as optimal. Subsequent optimization of cationic lipids revealed AX4-LNP formulation significantly enhanced antibody blocking rates (80.55% ± 2.06%) and IFN-γ responses (319.60 ± 45.23 SFC/10^5^ cells) compared to commercial vaccines. In swine trials, this vaccine maintained 54.76% ± 3.21% blocking rate at 120 days post-primary vaccination, surpassing the 40% positivity threshold.

In conclusion, this study innovatively combined the immunogenicity enhancement strategy based on self-assembling nanoparticles with cmRNA-LNP vaccine platform, providing novel approaches for the development of vaccines of CSFV.

## 2 Materials and methods

### 2.1 Cells

HEK293T cells and PK-15 cells were purchased from Cobier Biosciences (Nanjing, China) and BeNa Culture Collection (Xinyang, China). The cells were cultured in Gibco™ DMEM (Thermo Fisher Scientific, MA, USA) supplemented with 10% Gibco™ fetal calf serum (Thermo Fisher Scientific, MA, USA) and Gibco™ penicillin/streptomycin antibiotics (100 U/mL penicillin, 100 mg/mL streptomycin; Thermo Fisher Scientific, MA, USA). The cells were maintained at 37°C, 5% CO_2_, and 90% relative humidity.

### 2.2 The three-dimensional structure analysis of proteins encoded by cmRNA

The three-dimensional structure of proteins Encoded by cmRNA was modeled using the Alphafold2 2.3.1 server (https://github.com/deepmind/alphafold). The identified linear antigenic epitopes and different structural regions of the encoded protein were presented by the Pymol 3.1.3.1 software (https://www.pymol.org).

### 2.3 cmRNA preparations

In this study, the Clean-PIE strategy reported in the reference (35) was used to prepare cmRNA. The DNA sequences encompassing PIE elements, IRES, coding regions, and others were chemically synthesized and cloned into a pUC57 plasmid vector. Using the plasmid vector digested by XbaI as a template, *in vitro* transcription was carried out with the Purescribe T7 High Yield RNA Synthesis Kit (CureMed, Suzhou, China) to synthesize the cmRNA precursor. Subsequently, the cmRNA precursors were digested with DNase I (CureMed, Suzhou, China) for 15 minutes and then purified by the GeneJET RNA Purification Kit (Thermo Fisher Scientific, MA, USA). Then, the above-mentioned cmRNA precursor was added to a magnesium-containing buffer (50 mM Tris-HCl, pH 8.0; 10 mM MgCl2; and 1 mM DTT; Thermo Fisher) with 2 mM GTP, and the reaction was conducted at 55°C for 15 min. The reaction products were purified by HPLC based on a size-exclusion column with a particle size of 5 µm and a pore size of 1,000 Å (Sepax Technologies, Suzhou, China) on an SCG (Sepure Instruments) protein purification system (Sepure Instruments, Suzhou, China). The column was washed with RNase-free phosphate buffer (pH 6) at a flow rate of 15 mL/min. The purity and integrity of cmRNA were evaluated using an Agilent Bioanalyzer. For size analysis, agarose gel electrophoresis was performed with a linear ssRNA ladder (Thermo Fisher Scientific, MA, USA) as a molecular standard.

### 2.4 *In vitro* transfection of mRNA

To conduct cmRNA transfection, 1×10^5^ HEK293T cells were seeded per well into 24-well plates. Subsequently, following the manufacturer’s protocol, 500 ng of cmRNA was transfected into each well using the TransIT^®^-mRNA Transfection Kit (Mirus Bio, WI, USA).

After transfection, the transfected cells were incubated at 37°C in a 5% CO_2_ atmosphere for 24 h. After that, the culture supernatant was collected, and intracellular expression samples were prepared by lysing the cells with a cell lysis buffer. Subsequently, based on the 6×His protein tag fused to the C-terminus of the target protein, the protein expression levels in these samples were quantified using the His Tag ELISA Detection Kit (GenScript, Nanjing, China). The protein expression effects of each cmRNA were further confirmed by Western Blot. After electrophoresis, the gel was transferred to a PVDF membrane which was blocked with 5% skim milk in PBS at 37°C for 1 h, washed three times with PBST, and then incubated overnight at 4°C with primary antibodies (anti-E2 glycoprotein, 1000× dilution; National Center for Veterinary Culture Collection, Beijing, China). After washing, HRP-conjugated goat anti-mouse antibodies (2000× dilution, KPL, MD, USA) were applied at 37°C for 1h, followed by ECL detection using Enhanced ECL Chemiluminescence Detection Kit (Vazyme, Nanjing, China).

### 2.5 LNP encapsulation of cmRNA

The cmRNA was encapsulated using the LNP encapsulation method established in the references (36, 37) to prepare the cmRNA-LNP vaccine for immunization. The cmRNA was diluted in 10 mM citrate buffer (pH 4.0) to a final concentration of 200 μg/mL, the cationic lipid ALC0315 (JenKem, Beijing, China) or AX4 (Curemed, Suzhou, China) or SM102 (JenKem, Beijing, China), DSPC, cholesterol, and DMG-PEG were dissolved in ethanol. The cmRNA-LNP complex was prepared by microfluidics, with a volume ratio of mRNA to the mixture of lipids of 3:1, then the mixed product was purified by tangential flow filtration. The concentration and encapsulation rate of cmRNAs were measured by the Quant-it™ RiboGreen™ RNA Assay Kit (Thermo Fisher Scientific, MA, USA). The size of cmRNA-LNP complex was measured using dynamic light scattering on a Zetasizer Nano-ZS 300 (Malvern, UK) which is irradiated with a red laser.

### 2.6 Immunogenicity evaluation of mRNA-LNP vaccines in mice

To evaluate the immunogenicity of five cmRNA-LNP vaccines formulated via different sequence optimization strategies in mice, 30 female BALB/C mice aged 4–6 weeks were randomly divided into six equal-sized experimental groups of five mice each. Mice in Groups 1 to 5 were given an intramuscular injection with one of the five prepared cmRNA-LNP vaccines at a 10 μg dose per mouse. Mice in Group 6 served as the immunization control group, receiving the CSFV E2 protein recombinant baculovirus-inactivated vaccine (Strain WH-09, Wuhan Keqian Biologics, Wuhan, China), which is formulated with a water-in-oil emulsion adjuvant (oil adjuvant) and expresses E2 protein via a baculovirus system, at a 10 μg dose per mouse. All groups received a booster immunization 21 days after the initial one. At 21, 28, 35 and 49 days post-first immunization, blood was collected from the orbital vein to aseptically prepare serum. The CSFV antibody test kit (IDEXX, ME, USA) was used to measure the titers of CSFV-specific antibodies in the serum, with all procedures following the manufacturer’s instructions.

To evaluate the differences in immunogenicity among different LNPs, the cmRNA encoding E2-TMD-mi3 was encapsulated into three distinct LNPs, respectively. 20 female BALB/C mice aged 4–6 weeks were randomly divided into four equal-sized experimental groups of five mice each. Mice in Groups A to C were given an intramuscular injection with one of the three prepared cmRNA-LNP vaccines at a 10 μg dose per mouse. Mice in Group D serving as the commercial subunit vaccine immunization control group, were immunized following the manufacturer’s dosage guidelines. Serum samples from groups A to D were collected at the previously mentioned post-immunization time points and antibody titers were measured according to the procedures. On day 35 post-immunization, mice were euthanized via CO_2_ anesthesia. Splenocytes were then harvested under aseptic conditions, ground with pre-chilled sterile PBS, and filtered through a 70 μm cell strainer. ACK Buffer is used to remove red blood cells from cell suspensions, then the cell suspensions were centrifuged at 1500 rpm for 5 minutes. The cell pellet was resuspended in PBS and washed 2–3 times. Cell viability was assessed by trypan blue staining. Splenocytes were then cultured in Gibco™ RPMI 1640 supplemented with 10% Gibco™ fetal calf serum (Thermo Fisher Scientific, MA, USA) and Gibco™ penicillin/streptomycin (100 U/mL penicillin, 100 μg/mL streptomycin; Thermo Fisher Scientific, MA, USA) at 37°C in 5% CO_2_. The cellular immune level of immunized mice was measured using splenocytes with IFN-γ ELISpot assay kit (R&D Systems, MN, USA). Splenocytes were seeded into an ELISpot plate at a density of 1×10^5^ cells per well. For each cmRNA-LNP vaccine immunization group, 10 μg/mL of the E2 peptide pool (GenScript, Nanjing, China) was added as a stimulant. Phytohemagglutinin (PHA) was used as stimulant control, and DMSO was used as unstimulated control. After 72-hour stimulation, streptavidin-horseradish peroxidase substrate was added, and the number of spots in each well was counted using an ELISpot counter.

### 2.7 Immunogenicity evaluation of mRNA-LNP vaccines in pigs

To verify the immunoprotective effect of the mRNA-LNP vaccine-E2-TMD-mi3 based three distinct LNPs in pigs, 25 three-way crossbred pigs aged 28–40 days were randomly divided into five equal-sized experimental groups of five pigs each. Pigs in Groups I to III were given an intramuscular injection in the neck with one of the three prepared mRNA-LNP vaccines at a 50μg dose per pig. Pigs in Group 4 served as the immunization control group, receiving the CSFV E2 protein recombinant baculovirus-inactivated vaccine (Strain WH-09, Wuhan Keqian Biologics, Wuhan, China), at a 50 μg dose per pig. Group V, serving as the negative control, received an equal-volume injection of PBS. All pigs in the groups received a booster immunization at 21 days post-prime immunization.

Serum samples were collected from all groups at 21, 35, 60, 90, and 120 days post-prime immunization, and body weight was measured simultaneously. The CSFV antibody test kit (IDEXX, ME, USA) was employed to measure the titers of CSFV-specific antibodies in the serum, with all procedures strictly following the manufacturer’s instructions. Sera from immunized pigs at 35 and 120 days post-immunization (dpi) were inactivated at 56°C for 30 min. Serial dilutions were performed in 96-well plates: initial 4-fold dilution, followed by twofold serial dilutions up to 8192× (final volume: 50 µL/well). An equal volume of CSFV-SM strain (100 TCID_50_) was added to each well, and the mixture was incubated at 37°C for 1 h. Subsequently, 100 µL of PK-15 cells (2×10^4^ cells/well) were added, and plates were cultured at 37°C in a 5% CO_2_ incubator for 72 h. After culture, supernatants were discarded, and cells were washed 3× with PBS. Cells were fixed with 80% cold acetone at -20°C for 1 h, followed by 3× washes with PBST. Plates were then incubated with 50 µL of E2-specific monoclonal antibody WH303 (1:1000 dilution in PBS containing 10% calf serum) at 37°C for 1 h, washed 3× with PBST, and incubated with Goat anti-mouse Alexa Fluor 488 (1:200 dilution) at 37°C for 1 h. Final washes (3× with PBST) were performed before microscopy. Plates were observed under a fluorescence microscope to record positive wells with virus-infected cells (specific fluorescence). Neutralizing antibody titers (ND_50_) were determined according to the EU Classical Swine Fever Diagnostic Manual: the highest serum dilution at which 1 of 2 replicate wells exhibited specific fluorescence (infected cells) and the other did not.

### 2.8 Quantification and statistical analysis

Statistical analysis was performed using GraphPad Prism 9.5.1 (GraphPad Software). A two-way or one-way analysis of variance (ANOVA) was applied after confirming normality (Shapiro-Wilk test) and homogeneity of variances (Levene’s test). Tukey’s *post hoc* test was used for all pairwise comparisons, while Dunnett’s test was applied for comparisons against a control group. Statistical significance was defined as *P < 0.05, **P < 0.01, ***P < 0.001, or ****P < 0.0001.

## 3 Results

### 3.1 Design of cmRNA sequence combinations

**Figure 1:**
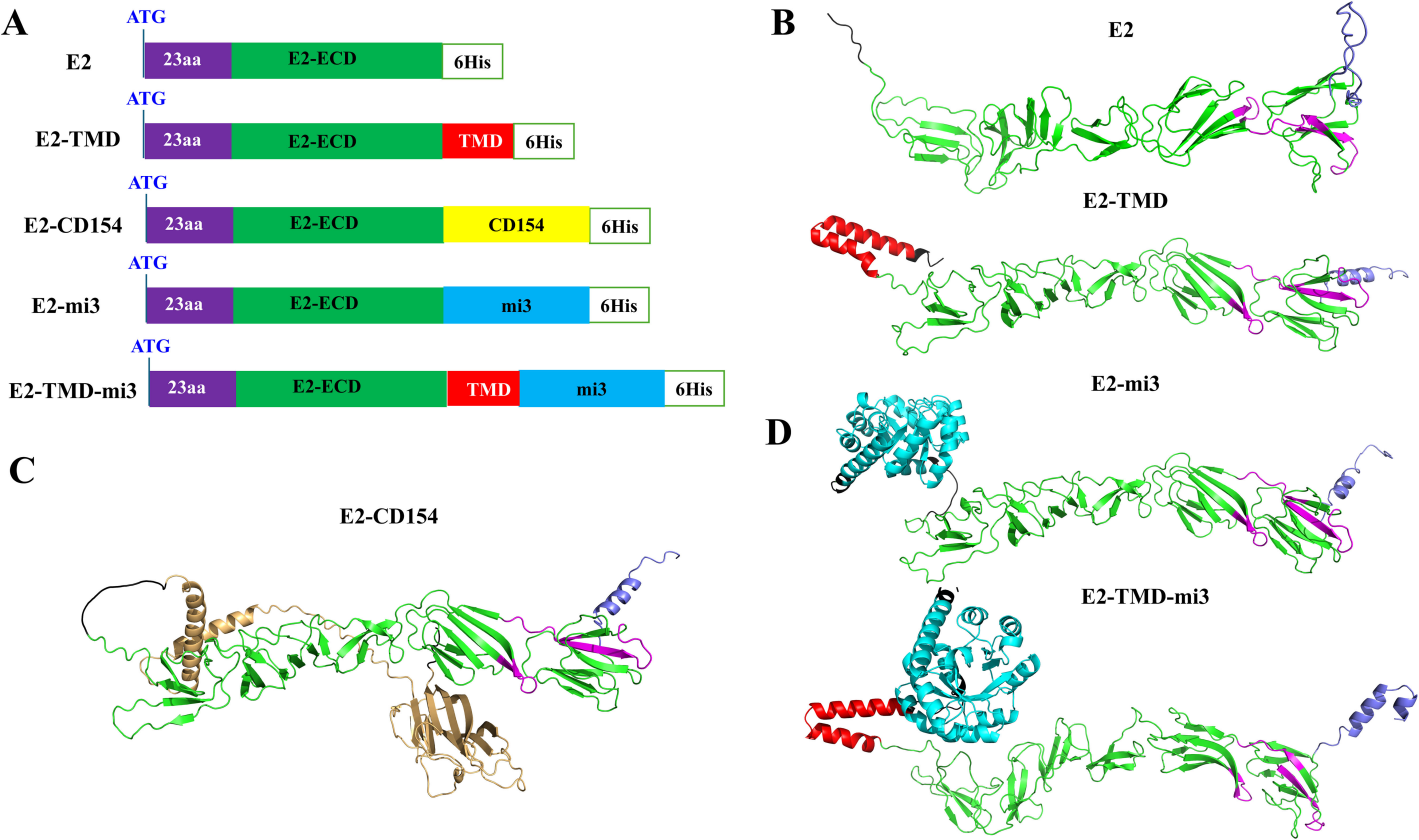
Design of mRNA sequence combinations. Schematic diagrams of the structures of the five designed sequences **(A)**. Predicted three-dimensional structures of proteins encoded by E2 and E2-TMD **(B)**. Predicted three-dimensional structures of proteins encoded by E2-CD154 **(C)**. Predicted three-dimensional structures of proteins encoded by E2-mi3 and E2-TMD-mi3 **(D)**. The ECD is highlighted in green, the TMD in red, the linker and 6×His tag in black, the identified antigenic epitopes in pink, the signal peptide in purple, CD154 in yellow, and the mi3 in blue.

As the glycoprotein with the best immunogenicity in classical swine fever virus, the E2 protein serves as an important target for vaccine development. Therefore, in this study, based on the coding sequence of the E2 glycoprotein, a series of optimized E2 glycoprotein sequences (GenBank accession number: AY663656.1) were designed by genetically fusing the molecular adjuvant (CD154, a member of the tumor necrosis factor superfamily, GenBank accession number: NP_035746.2) or self-assembled nanoparticle mi3 to the E2 glycoprotein C-terminus. A total of five different coding sequences were designed. To facilitate protein expression detection, a 6×His tag coding sequence was genetically incorporated at the C-terminus of all designed sequences (**Figure 1A**). Among them, the coding sequence E2 only retained the signal peptide of the E2 glycoprotein and the extracellular domain (ECD) where neutralizing antigenic epitopes are most concentrated. The coding sequence E2-TMD retained the above two structural units and the transmembrane domain (TMD) of the E2 protein. Based on the E2 sequence, the coding sequences E2-CD154 and E2-mi3 were generated by genetically fusing the molecular adjuvant CD154 and self-assembled nanoparticle mi3 to its C-terminus. These components were connected via a double GGGGS flexible linker to ensure that the two parts flanking the linker could fold independently into correct spatial conformations. Based on the E2-TMD sequence, a coding sequence named E2-TMD-mi3 was designed by fusing the self-assembled nanoparticle mi3 to its C-terminus, following the same strategy as described earlier. To ensure that the fusion of CD154 or mi3 does not disrupt the native folding of the E2 glycoprotein and masks its original antigenic epitopes, this study used AlphaFold to predict the three-dimensional structures of the proteins encoded by the five sequences. The three-dimensional structures of the proteins encoded by E2 and E2-TMD were substantially identical, with the key difference being the presence of an α-helix formed by the TMD (highlighted in red) at the C-terminus of E2-TMD (**Figure 1B**). The three-dimensional structure of the protein encoded by the E2-CD154 sequence formed two independent folded units. The CD154 (highlighted in yellow) did not disrupt the folding of the E2 protein, as no significant alterations were observed in the E2 tertiary structure. Additionally, the antigenic epitopes (highlighted in pink) previously identified (38) in the ECD (highlighted in green) remained fully exposed (**Figure 1C**). Similarly, both E2-mi3 and E2-TMD-mi3 formed two independent folded units, with mi3 (highlighted in blue) and E2/E2-TMD each folding into their respective spatial conformations (**Figure 1D**). Thus, fusion expression of the molecular adjuvant CD154 or self-assembling nanoparticles mi3 did not alter the native structure of E2 glycoprotein, ensuring proper presentation of both conformational and linear epitopes. This is crucial for our subsequent development of the cmRNA-LNP vaccine.

### 3.2 Verification of the cmRNA sequence combinations

**Figure 2:**
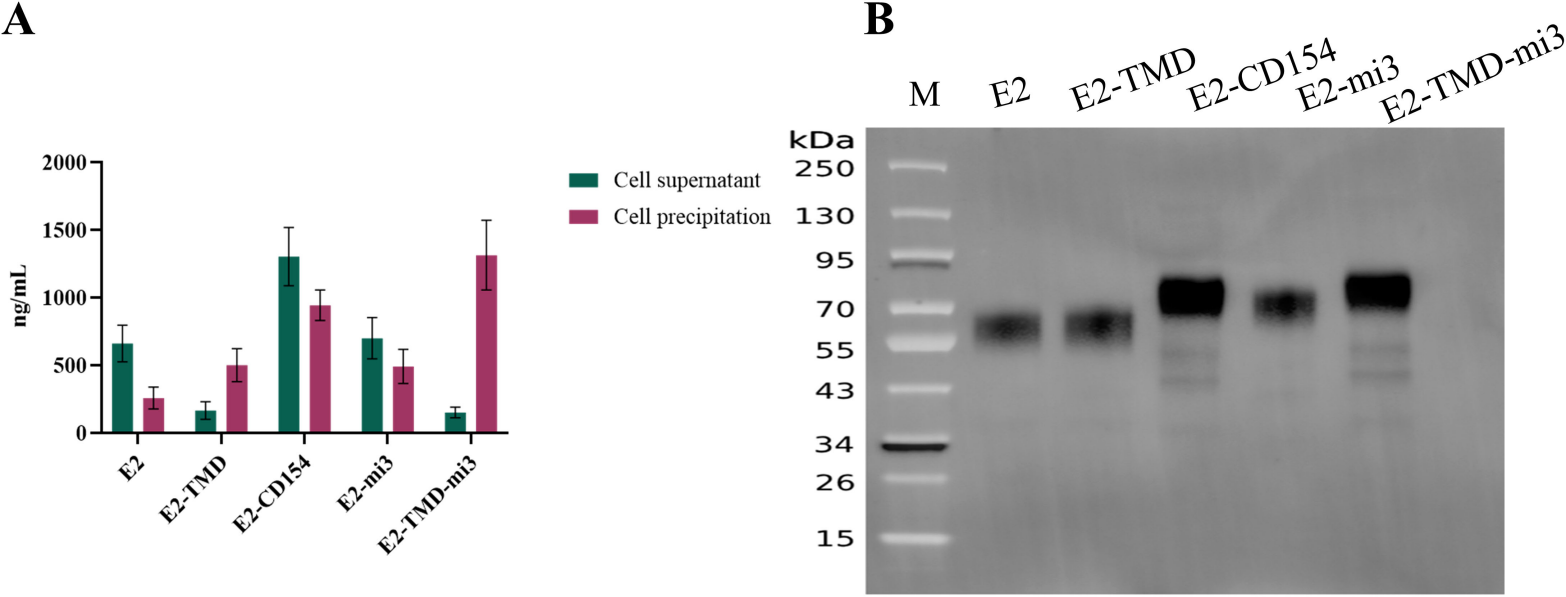
Verification of the mRNA sequence combinations. Quantification of protein expression levels of five cmRNAs in different fractions 24 h post-transfection **(A)**. Western Blotting analysis of cell lysates from five cmRNAs 24 h post-transfection **(B)**. Lane M, protein ladder.

In this study, Capillary electrophoresis (CE, using a 3100 Genetic Analyzer with an internal standard, 15 kV, 60 min), and *in vitro* transfection experiments (detection of protein expression levels based on His-Tag ELISA and Western blotting based on an anti-E2 protein monoclonal antibody) were used to further evaluate the quality of the cmRNA prepared based on the above five coding sequences. A Capillary electrophoresis analysis showed that the main peak of cyclized products was consistent with expectations, with purity ranging from 92% to 97% (**Table 1**). This indicates that the cmRNA prepared in this study has good quality and can be used for subsequent vaccine preparation. Building on these results, we further confirmed whether the five cmRNAs described earlier could be properly expressed *in vitro*. At 24 h post-transfection, the E2, E2-CD154, and E2-mi3 proteins were mainly expressed in the culture supernatant, with concentrations of 660.87 ± 60.15 ng/mL, 1302.21 ± 96.33 ng/mL, and 699.87 ± 68.49 ng/mL respectively. In contrast, the E2-TMD and E2-TMD-mi3 proteins had higher concentrations in the cell lysates, at 501.18 ± 54.63 ng/mL and 1313.11 ± 115.36 ng/mL respectively (**Figure 2A**). This might be related to the fact that the latter two proteins retained the transmembrane domain. Moreover, the Western Blotting analysis of the cell lysates 24 h post-transfection further demonstrated that the band positions were consistent with the expectations (**Figure 2B**). In conclusion, the cmRNA prepared in this study can be used for the subsequent formulation of cmRNA-LNP vaccines.

**Table 1:** Results of capillary electrophoresis analysis for 5 cmRNA.

Name	Main peak size (nt)	Percentage (%)
E2	1968	97
E2-TMD	2070	96
E2-CD154	2640	94
E2-mi3	2613	95
E2-TMD-mi3	2715	92

### 3.3 Verification of the immune effects of sequence combinations in mice

To further validate the immunogenicity of the five prepared cmRNAs in mice, this study encapsulated the above five cmRNAs using ACL0315-LNP and characterized the particle size and polydispersity index (PDI) of the cmRNA-LNP complexes. The five cmRNA-LNP formulations exhibited high encapsulation efficiency (93.4%-96.1%) and uniform particle sizes (92.4-96.7 nm, PDI 0.15-0.2), indicating robust formulation quality (**Table 2**). These properties are essential for efficient cellular delivery and immune activation.

**Table 2:** Characterization of key parameters of cmRNA-LNP complexes.

Name	Size (nm)	PDI	Encapsulation percentage (%)
E2/ALC0315	96.7	0.16	93.7
E2-TMD/ALC0315	95.5	0.20	96.1
E2-CD154/ALC0315	96.3	0.15	94.5
E2-mi3/ALC0315	92.4	0.21	93.4
E2-TMD-mi3/ALC0315	95.6	0.20	94.1

**Figure 3:**
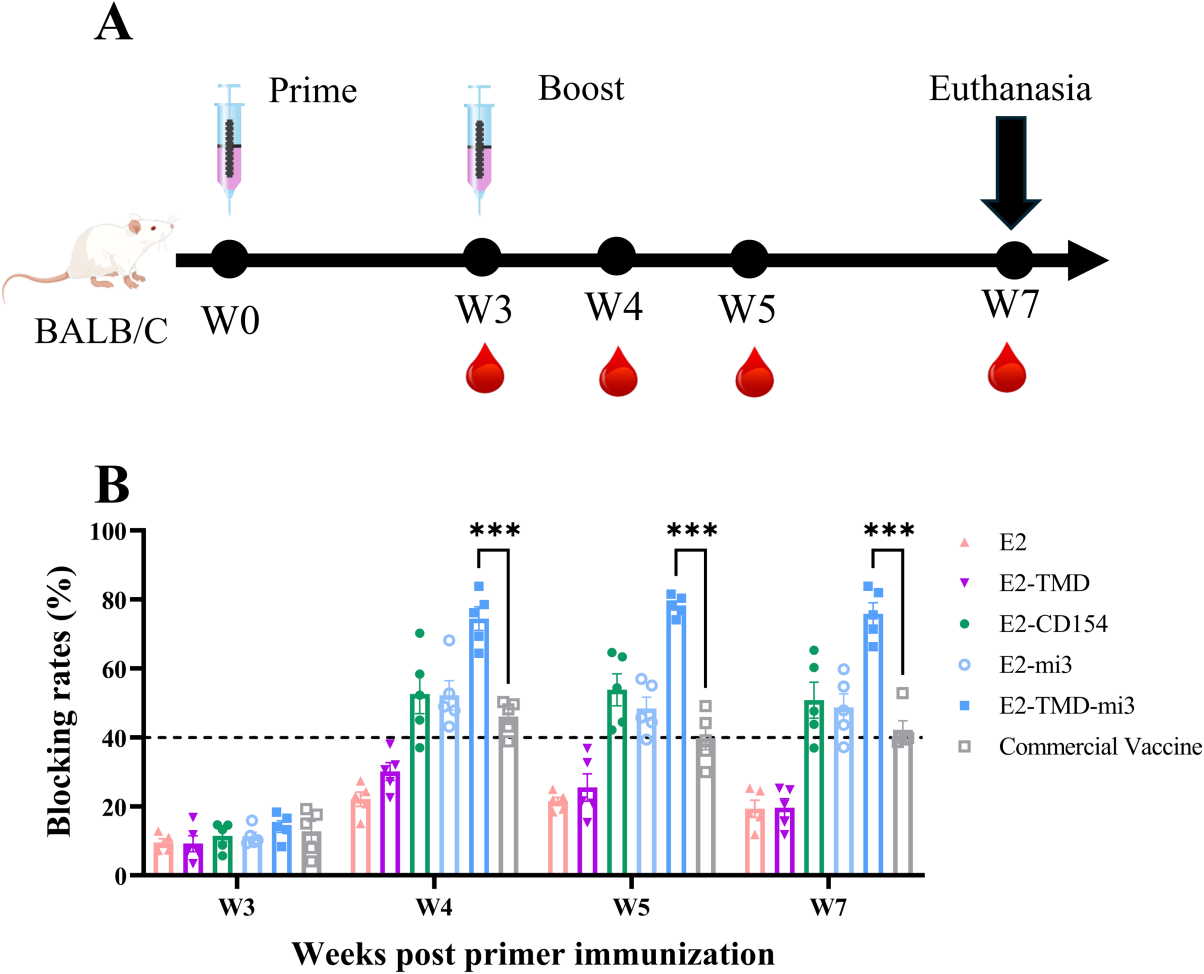
Verification of the immune effects of sequence combinations in mice. Schematic diagram of the cmRNA-LNP (ALC0315) vaccination in mice **(A)**. Changes in serum antibody blocking rates of each cmRNA-LNP vaccine and the commercial E2 protein subunit vaccine at 3, 4, 5, 7 weeks post-immunization (Serum dilution 1:100, n=5 mice per group) **(B)**. Statistical differences in mean antibody blocking rates were analyzed by two-way ANOVA followed by Dunnett’s multiple comparisons test (***P <0.001 vs. commercial E2 subunit vaccine group). Non-significant differences (*P*≥0.05) were omitted from the figure. All data are presented as mean ±SEM (Standard Error of the Mean).

Following the experimental protocol (**Figure 3A**), we immunized mice (n=5 per group) and collected their blood to measure serum antibody blocking rates, aiming to evaluate the immunogenicity of the five cmRNA-LNP vaccines. Before the booster immunization, antibody locking rates in all immunized groups were below the positivity threshold (<40%, set according to the manufacturer’s specifications). One week after the booster, all cmRNA-LNP vaccine groups except E2 and E2-TMD seroconverted, while E2 and E2-TMD groups remained non-responsive. Statistical analysis revealed that only the E2-TMD-mi3 induced significantly higher blocking rates compared to the commercial E2 subunit vaccine group at 4, 5, and 7 weeks after the primary immunization. Specifically, at 4 weeks, the blocking percentage in the E2-TMD-mi3 group was 74.46% ± 3.43%, while that in the commercial E2 subunit vaccine was 46.02% ± 2.18% (two-way ANOVA, *p* < 0.001); at 5 weeks, the rates were 78.25% ± 1.32% and 39.74% ± 3.29% respectively (*p* < 0.001); at 7 weeks, they were 75.82% ± 3.26% and 42.18% ± 2.69% respectively (p < 0.001).Conversely, the E2 induced significantly lower mean blocking rates than the commercial vaccine group at 5 weeks post-immunization (two-way ANOVA, *p* < 0.05), and both E2 and E2-TMD groups exhibited significantly reduced blocking percentage at 7 weeks post-immunization (two-way ANOVA, *p* < 0.01) (**Figure 3B**). Collectively, among the five cmRNA-LNP vaccines developed in this study, E2-TMD-mi3 exhibited the best immunogenicity.

### 3.4 Comparison of the immunization effects of different LNPs in mice

Given the substantial influence of LNP composition on the immunogenicity of cmRNA-LNP vaccines, this study evaluated the differences in immune responses of cmRNA-LNP vaccines formulated with three different LNPs (AX4, ACL0315, and SM102) at both humoral and cellular levels. Similarly, the particle size, PDI, and encapsulation efficiency of the cmRNA-LNP complexes formulated with different LNPs were characterized following preparation. The three cmRNA-LNP formulations exhibited high encapsulation efficiency (93.7%–97.7%) and uniform particle sizes (86.1–103.3 nm, PDI 0.18–0.25), indicating robust formulation quality (**Table 3**).

**Table 3:** Characterization of key parameters of cmRNA-LNP complexes.

Name	Size (nm)	PDI	Encapsulation percentage (%)
E2-TMD-mi3/AX4	86.1	0.25	93.7
E2-TMD-mi3/SM102	97.5	0.18	96.1
E2-TMD-mi3/ALC0315	103.3	0.19	94.5

**Figure 4:**
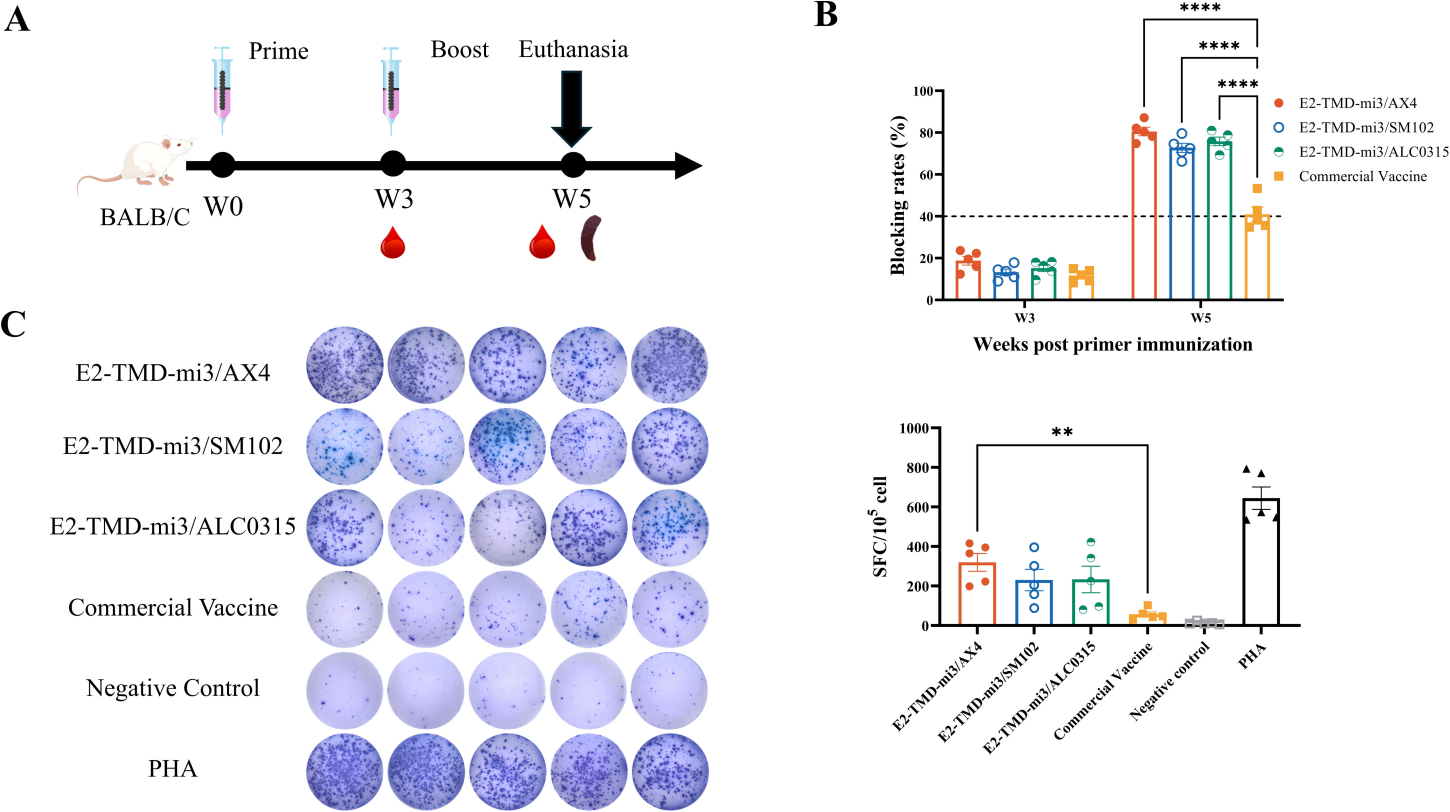
Comparison of the immunization effects of different LNPs in mice. Schematic diagram of the cmRNA-LNP vaccination in mice **(A)**. Changes in serum antibody blocking percentage of each cmRNA-LNP vaccine and the commercial E2 protein subunit vaccine at 3, 5weeks post-immunization (Serum dilution 1:100, n=5 mice per group) **(B)**. IFN-γ ELISpot assay of each cmRNA-LNP vaccine and the commercial E2 protein subunit vaccine at 5 weeks post-immunization (DMSO was used as the negative control stimulant, while PHA served as the positive control stimulant., n=5 mice per group) **(C)**. Statistical differences in mean antibody blocking percentages were analyzed by one-way or two-way ANOVA followed by Dunnett’s multiple comparisons test (**P <0.01, ****P <0.0001 vs. commercial E2 group). Non-significant differences (*P*≥0.05) were omitted from the figure. All data are presented as mean ± SEM (Standard Error of the Mean).

Mice were immunized according to the protocol (**Figure 4A**). Serum samples were collected at 3 weeks post-primary immunization. At 5 weeks, both serum and splenocytes were harvested. The serum samples were used to evaluate humoral immune responses (antibody blocking rates), while the splenocytes were used for the cellular immune responses assessment through IFN-γ ELISpot assay. Consistent with previous findings, serum antibody neutralization rates in all immunized groups remained below the detection threshold at 3 weeks post-primary immunization. However, two weeks after the booster, all mice in the cmRNA-LNP vaccine groups showed blocking rates above the detection threshold, which were significantly higher than those of the commercial vaccine group (80.56% ± 2.06%, 72.76% ± 2.19%, 75.82% ± 2.03% vs. 41.00% ± 3.38%; two-way ANOVA, p<0.0001). No significant differences were observed among the cmRNA-LNP vaccine groups (**Figure 4B**). IFN-γ ELISpot assay showed that AX4-LNP formulated E2-TMD-mi3 induced 5.63-fold higher antigen-specific T cell responses (319.60 ± 45.23 SFC/10^5^ cells) than the commercial E2 subunit vaccine (56.8 ± 12.50 SFC/10^5^ cells) at 2 weeks post-boost (one-way ANOVA, *p*<0.01). Although no statistically significant differences were observed between cmRNA-LNP vaccines formulated with other LNPs and the commercial vaccine, SM102-LNP and ALC0315-LNP induced responses 4.05-fold (230.20 ± 53.98 SFC/10^5^ cells) and 4.11-fold (233.60 ± 66.95 SFC/10^5^ cells) higher antigen-specific T cell responses than the commercial vaccine, respectively. (**Figure 4C**). These data demonstrate that cmRNA-LNP vaccines formulated with different LNPs all exhibited superior immunogenicity compared to the commercial E2 subunit vaccine, with AX4-LNP yielding the highest efficacy.

### 3.5 Comparison of the immunization effects of different LNPs in pigs

**Figure 5:**
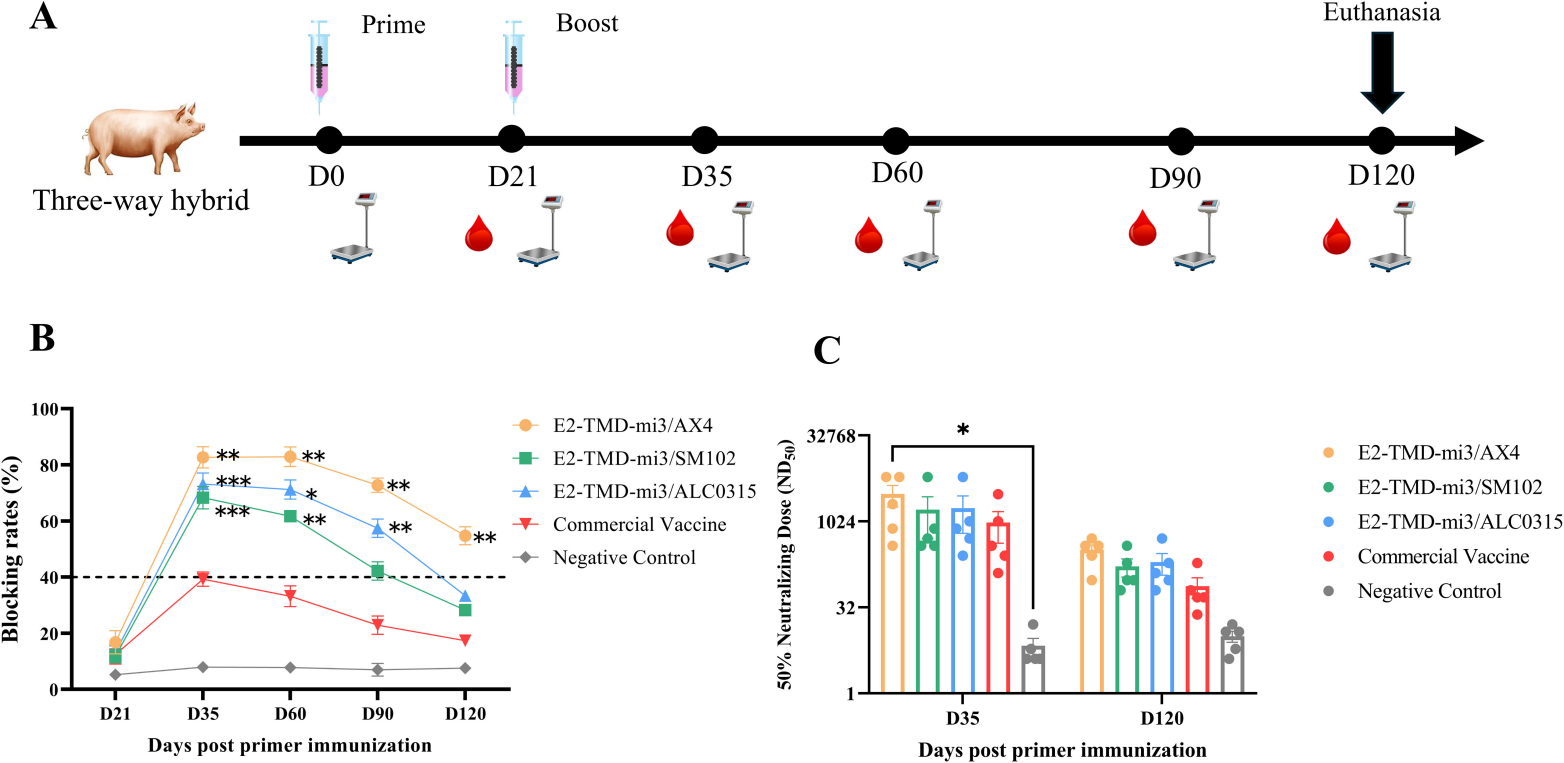
Comparison of the immunization effects of different LNPs in pigs. Schematic diagram of the cmRNA-LNP vaccination in pigs **(A)**. Changes in serum antibody blocking percentage of each cmRNA-LNP vaccine and the commercial E2 protein subunit vaccine at 21, 35, 60, 90, 120 days post-immunization (Serum dilution 1:100, n=5 pigs per group) **(B)**. Serum neutralizing titer assays at 35 days and 120 days after the first immunization (n=5 pigs per group) **(C)** Statistical differences in mean antibody blocking percentages were analyzed by two-way ANOVA followed by Dunnett’s multiple comparisons test (*P <0.05, **P <0.01, ***P <0.001 vs. commercial E2 group). Non-significant differences (*P*≥0.05) were omitted from the figure. All data are presented as mean ± SEM (Standard Error of the Mean).

To compare the immunogenicity and safety profiles of three LNP-encapsulated cmRNA vaccines (AX4-LNP, ALC0315-LNP, and SM102-LNP) with a commercial E2 subunit vaccine in pigs (n=5 per group), animals were immunized and serum samples were collected at days 21, 35, 60, 90 and 120 for antibody analysis, while body weight was recorded weekly to assess systemic tolerability (**Figure 5A**). Like the results of mouse experiments, 21 days after the primary immunization, the antibody blocking rates in all immunization groups were below the positive threshold. Seroconversion occurred 14 days after the booster immunization. However, in the commercial vaccine group, only one pig had a blocking rate above the positive threshold, while the rest remained below the threshold and subsequently turned seronegative (**Figure 5B**). 14 days after booster immunization, antibody blocking rates in the cmRNA-LNP vaccine groups (E2-TMD-mi3/AX4, E2-TMD-mi3/ALC0315, and E2-TMD-mi3/SM102) were significantly higher than those in the commercial vaccine group (two-way ANOVA, p < 0.01 and p < 0.001), reaching 82.72% ± 3.78%, 73.21% ± 3.95%, and 68.36% ± 4.04% respectively (**Figure 5B**). For the E2-TMD-mi3/AX4 vaccine formulation, antibody blocking rates remained at 82.94% ± 3.55% 60 days post-primary immunization. Although antibody levels began declining at 90 and 120 days post-primary immunization, they remained above the detection threshold and significantly higher than those of the commercial vaccine group (two-way ANOVA, p < 0.01). In contrast, both E2-TMD-mi3/ALC0315 and E2-TMD-mi3/SM102 formulations became seronegative by 120 days post-primary vaccination. The results related to the neutralizing titers of antibodies in each group showed that the antibody neutralizing titers of each mRNA-LNP group at 35 days and 120 days after primary immunization were comparable to those of the commercial vaccine group, with no significant difference between groups (two-way ANOVA, p > 0.05). Only 35 days after the first immunization, the neutralizing antibody titer of the E2/TMD-mi3/AX4 group was significantly higher than that of the negative control group (two-way ANOVA, p < 0.01) (**Figure 5C**). Throughout the entire experimental period, no statistically significant differences (two-way ANOVA, p ≥ 0.05) in weight gain rates were observed between vaccinated pigs and the negative control group (immunized with sterile PBS only). This indicates that the three cmRNA-LNP vaccines formulated with different LNPs (AX4, ALC0315, and SM102) were all safe and did not compromise the production performance of immunized animals (**Supplementary Figure S1**). Overall, the three cmRNA-LNP vaccines demonstrated superior immunogenicity compared to the commercial vaccine and exhibited favorable safety profiles. Among them, the AX-4 LNP-encapsulated cmRNA-LNP vaccine showed the optimal performance, inducing higher levels of humoral immunity and providing prolonged antibody persistence.

## 4 Discussion

At present, CSFV still poses a significant threat to the global swine industry. Although the cases of CSFV in China are sporadically reported, efforts are still required to achieve the elimination of CSFV (10, 39). E2 subunit vaccines with DIVA capability continue to be crucial instruments in the fight to eradicate CSFV. Nevertheless, the high production costs coupled with suboptimal immunogenicity have restricted their further application (13). To address these challenges in CSFV elimination, next-generation vaccine platforms requiring both biosafety and high immunogenicity are urgently needed. CircRNA vaccines have emerged as a promising platform in vaccine development against major infectious diseases, including SARS-Cov-2 (40, 41), and monkeypox virus (42), which combines the biosafety advantages of traditional subunit vaccines with enhanced production efficiency. Therefore, for the first time, we developed the cmRNA-LNP vaccine by integrating an immunogenicity enhancement strategy into the cmRNA-LNP platform, resulting in a vaccine that exhibits better immunogenicity than commercial vaccines.

Herein, we designed a coding sequence for E2-mi3 nanoparticles by fusing the mi3 self-assembling particles to the C-terminus of the E2 protein to enhance the immunogenicity of the E2 protein. This strategy has been extensively employed to bolster the immunogenicity of the E2 protein, yielding immune responses that outperform those elicited by subunit vaccines (24, 43). When encapsulated with ALC0315-LNP, the E2-TMD-mi3 vaccine induced significantly higher antibody levels in mice compared to the commercial vaccine after booster vaccination (p < 0.01), with a maximum antibody blocking rate of 78.25%± 1.32%. Although the E2-CD154 vaccine incorporating a molecular adjuvant strategy also elicited higher antibody levels than the commercial product, no statistically significant difference was observed. Notably, the E2-TMD-mi3vaccine designed using the mi3 self-assembled particle strategy induced significantly higher antibody levels in mice compared to the E2-mi3 formulation and CD-154 formulation. *In vitro* expression results of this study showed that E2-TMD-mi3 was predominantly expressed in cell pellets, while a significant portion of E2-mi3 was secreted into the culture supernatant. This is likely due to the presence of the TMD, which enhances the cell membrane localization of E2-TMD-mi3. Such enhanced membrane localization may enable E2-TMD-mi3 to localize to the surface of antigen-presenting cells (APCs) at higher density, thereby increasing the antigen density of E2-TMD-mi3 and avoiding extensive secretion and subsequent dilution of antigens (44). This may be a critical factor contributing to the immunogenicity difference between E2-TMD-mi3 and E2-mi3. Similar changes in antigen immunogenicity caused by cellular localization differences have been observed in other mRNA vaccine studies (45), indicating that the subcellular localization of antigen proteins delivered by mRNA significantly impacts their immunogenicity. Relevant findings suggest that, compared to soluble antigens secreted extracellularly, membrane-localized antigens can directly activate B cells via B cell receptors (BCRs) by enhancing antigen presentation density. This may explain why E2-TMD-mi3 exhibits superior immunogenicity, although the specific mechanisms require further investigation in subsequent experiments. This suggests that while the TMD is not a region rich in epitopes, it still influences the immunogenicity of the E2 protein. The underlying mechanisms require further investigation. In contrast to the three constructs mentioned above, both E2 and E2-TMD failed to induce antibody levels in immunized mice exceeding those of the commercial vaccine group. Moreover, 4 weeks after booster vaccination, their antibody levels were significantly lower than those of the commercial vaccine group (p < 0.01). From a mechanistic perspective, the rational design of the E2-TMD-mi3 fusion coding sequence enabled two critical improvements (1): self-assembling nanoparticles enhancing molecular diameter versus monomeric E2, and (2) high-density epitope display. The molecular diameter of an antigen is a crucial factor influencing the uptake efficiency of antigen-presenting cells (APCs) (46). The larger molecular diameter of the E2-TMD-mi3 nanoparticles enables them to be presented and taken up more efficiently (46). Moreover, the higher antigen density enables E2-TMD-mi3 nanoparticles to cross-link with B-cell receptors (BCRs) more efficiently (47). This, in turn, promotes the activation of B cells and induces the body to produce higher levels of antibodies. Consequently, within 120 days after immunizing pigs, the cmRNA-LNP vaccine encapsulated with AX4-LNP and based on coding sequence for E2-TMD-mi3 nanoparticles induced the production of antibodies at levels significantly higher than those induced by commercial subunit vaccines (p < 0.01), with an antibody blocking rate of 54.76% ± 3.21%. Similarly, cmRNA-LNP vaccines encapsulated with ALC0315-LNP and SM102-LNP also induced more prolonged antibody persistence compared to the commercial vaccine in pigs.

Beyond antigen design optimization, the delivery system of cmRNA was identified as another critical determinant of cmRNA vaccine efficacy (31). As a crucial component of LNP, ionizable cationic lipids (ICL) exert a significant impact on immunogenicity of RNA vaccines (48). Therefore, in this study, we also compared the immunogenicity of cmRNA-LNPs vaccines formed by different ICL. The relevant results show that only the cmRNA-LNP vaccine formed by the ICL AX4 induced significantly higher cellular immune responses in immunized mice compared to commercial subunit vaccines (319.6 ± 45.23 SFC/10^5^ cells, p < 0.01). In pigs, serum antibody blocking rates remained above detection thresholds even 120 days post-primary immunization, providing superior immunogenicity compared to commercial vaccines. However, when encapsulating the same cmRNA, the antibodies induced by the other two LNPs could only last until 90 days after the primary immunization, and their antibody levels were lower than those of AX4-LNP throughout the entire antibody persistence period. Whether the tail of the ICLs has branched chains and whether the connecting bonds in the tail are easily degradable are important factors affecting the mRNA delivery efficiency and the release rate in endosomes, which in turn influences the mRNA expression level (49). This is an important factor leading to the differences in the immunization effects of mRNA-LNP vaccines prepared based on different ICL. However, in this study, the tail of AX-4 used has four branched chains, each containing a butyl octanoic acid moiety and eight ester bonds that are easily degradable at its tail (36). This endows the AX4-LNP with better mRNA delivery efficiency. As a result, when delivering the same cmRNA, the improved delivery efficiency leads to higher intracellular mRNA translation, resulting in a stronger immune response and enhanced vaccine efficacy. Notably, recent reports indicate that the AX4-LNP-formulated cmRNA-LNP vaccine not only remains effective after six months of storage at 4°C but also maintains stability through multiple freeze-thaw cycles, while exhibiting significantly lower hepatorenal toxicity. These attributes endow the vaccine developed in this study with promising clinical potential for practical applications (36).

Compared with the traditional eukaryotic expression system production platform, the RNA vaccines production platform has multiple advantages. RNA vaccines production platform with cells-free production can be produced more quickly and efficiently compared to traditional foreign protein expression systems. Just a single 5-liter bioreactor can produce millions of doses of vaccine in a single reaction (50). Compared with mRNA, cmRNA not only has a higher level of protein expression, but can also be stored and transported at room temperature and can be repeatedly frozen and thawed several times (32). This further reduces the transportation cost of vaccines, which accounts for most vaccine costs. Therefore, different from other studies on E2 subunit vaccines based on mi3 self-assembled nanoparticles (24, 43), this study used an cmRNA-LNP vaccine platform to deliver the E2-TMD-mi3 coding sequence. Although cmRNA - LNP vaccines demonstrate robust immunoprotective efficacy, residual double -stranded pre-RNA and introduced PIE elements from the cmRNA production process, among other nucleic acid molecules, can act as host innate immunity stimulants. This may lead to immunological side effects in vaccinated animals, potentially compromising vaccine safety (31). Therefore, this study employed the Clean-PIE strategy for circular mRNA production. By leveraging coding sequences as PIE elements to minimize the introduction of non-coding sequences and further enhancing cmRNA purity through size - exclusion chromatography-based HPLC, the safety of cmRNA-LNP vaccines was ensured, which is conducive to their widespread adoption in livestock farms.

Additionally, recent studies have shown that mRNA-LNP vaccines can induce the secretion of type I IFN and pro-inflammatory signals, thereby initiating downstream adaptive immune responses (51). Moreover, LNPs themselves can modulate the gene expression of macrophages and dendritic cells (51, 52). As a key negative regulator, TRIM29 deficiency regulates the PERK-mediated ER stress pathway, thereby enhancing local antiviral capacity and conferring protection against both lethal influenza infection and viral myocarditis (53, 54). PARP9, likewise serving as a noncanonical RNA virus sensor, can enhance type I IFN (55). Given TRIM29’s role as a negative regulator and PARP9’s antiviral function, therefore, we speculate that the mRNA-LNP developed in this study may further enhance protective immunity by transiently downregulating TRIM29 or upregulating PARP9 expression. In subsequent research, we will directly assess the expression kinetics of TRIM29 and PARP9 following vaccination to deeply elucidate the mechanisms by which the mRNA-LNP delivering the E2-TMD-mi3 coding sequence elicits robust immunogenicity.

Overall, this study engineered a cmRNA-LNP vaccine with superior immunogenicity to commercial subunit vaccines, which is more suitable for large-scale production. When paired with an ELISA kit designed for detecting anti-E^rns^ protein antibodies, this vaccine enables the effective implementation of the DIVA strategy. This not only provides a robust and reliable approach but also serves as a powerful tool in the efforts to eradicate CSFV. Furthermore, this research expands the research horizons by demonstrating the practical feasibility of cmRNA-LNP vaccines in the development of porcine virus vaccines. Moreover, it presents innovative and promising strategies that can potentially be adapted and applied to the development of vaccines against a wide array of other pathogens. Despite these significant achievements, constrained by biosafety regulations, this study was unable to verify the immunoprotective effect of the vaccine through a challenge experiment. Whether the vaccine prepared in this study can provide cross-protection against different genotypes of CSFV remains an open question. Additionally, although this study validated the promising immunogenicity of the cmRNA-LNP vaccine delivering the E2-TMD-mi3 coding sequence in both mouse and pig models, it was limited by experimental conditions and time constraints. We were unable to verify the *in vitro* and *in vivo* expression efficiency under different LNP deliveries, assess whether it could elicit high cellular immune responses in pigs comparable to those in mice, or provide an in-depth explanation of the mechanisms underlying its good immunogenicity. In future research, it would be necessary to explore alternative experimental models or settings that comply with biosafety regulations to verify the immunoprotective effect and cross - protection of the vaccine. Additionally, systematic studies on the stability of the cmRNA-LNP vaccine under various conditions are needed to optimize its storage and transportation.

## Data Availability

The datasets presented in this study can be found in online repositories. The names of the repository/repositories and accession number(s) can be found below: https://www.ncbi.nlm.nih.gov/genbank/, AY663656.1; https://www.ncbi.nlm.nih.gov/genbank/, NP_035746.2.

## References

[1] Ji W Guo Z Ding NZ He CQ. Studying classical swine fever virus: making the best of a bad virus. Virus Res. (2015) 197:35–47. doi: 10.1016/j.virusres.2014.12.006 25510481

[2] Moennig V Floegel-Niesmann G Greiser-Wilke I. Clinical signs and epidemiology of classical swine fever: A review of new knowledge. Vet J. (2003) 165:11–20. doi: 10.1016/S1090-0233(02)00112-0 12618065

[3] King AM Lefkowitz E Adams MJ Carstens EB. Virus Taxonomy: Ninth Report of the International Committee on Taxonomy of Viruses. Elsevier (2011). Available online at: https://www.researchgate.net/publication/232743502_Virus_Taxonomy_Ninth_Report_of_the_International_Committee_on_Taxonomy_of_Viruses

[4] Thiel HJ Stark R Weiland E Rümenapf T Meyers G. Hog cholera virus: molecular composition of virions from a pestivirus. J Virol. (1991) 65:4705–12. doi: 10.1128/jvi.65.9.4705-4712.1991 PMC248926 1870198

[5] Bintintan I Meyers G. A new type of signal peptidase cleavage site identified in an rna virus polyprotein. J Biol Chem. (2010) 285:8572–84. doi: 10.1074/jbc.M109.083394 PMC2838279 20093364

[6] Gottipati K Ruggli N Gerber M Tratschin JD Benning M Bellamy H. The structure of classical swine fever virus N(Pro): A novel cysteine autoprotease and zinc-binding protein involved in subversion of type I interferon induction. PloS Pathog. (2013) 9:e1003704. doi: 10.1371/journal.ppat.1003704 24146623 PMC3798407

[7] Heimann M Roman-Sosa G Martoglio B Thiel HJ Rümenapf T. Core protein of pestiviruses is processed at the C terminus by signal peptide peptidase. J Virol. (2006) 80:1915–21. doi: 10.1128/jvi.80.4.1915-1921.2006 PMC1367156 16439547

[8] Elbers K Tautz N Becher P Stoll D Rümenapf T Thiel HJ. Processing in the pestivirus E2-ns2 region: identification of proteins P7 and E2p7. J Virol. (1996) 70:4131–5. doi: 10.1128/jvi.70.6.4131-4135.1996 PMC190302 8648755

[9] Fan J Liao Y Zhang M Liu C Li Z Li Y. Anti-classical swine fever virus strategies. Microorganisms. (2021) 9(4):761. doi: 10.3390/microorganisms9040761 33917361 PMC8067343

[10] Luo Y Li S Sun Y Qiu H-J. Classical swine fever in China: A minireview. Vet Microbiol. (2014) 172:1–6. doi: 10.1016/j.vetmic.2014.04.004 24793098

[11] de Smit AJ. Laboratory diagnosis, epizootiology, and efficacy of marker vaccines in classical swine fever: A review. Vet Q. (2000) 22:182–8. doi: 10.1080/01652176.2000.9695054 11087126

[12] Wei Q Liu Y Zhang G. Research progress and challenges in vaccine development against classical swine fever virus. Viruses. (2021) 13(3):445. doi: 10.3390/v13030445 33801868 PMC7998128

[13] Huang YL Deng MC Wang FI Huang CC Chang CY. The challenges of classical swine fever control: modified live and E2 subunit vaccines. Virus Res. (2014) 179:1–11. doi: 10.1016/j.virusres.2013.10.025 24211665

[14] Borca MV Holinka LG Ramirez-Medina E Risatti GR Vuono EA Berggren KA. Identification of structural glycoprotein E2 domain critical to mediate replication of classical swine fever virus in sk6 cells. Virology. (2019) 526:38–44. doi: 10.1016/j.virol.2018.10.004 30340154

[15] Vuono EA Ramirez-Medina E Holinka LG Baker-Branstetter R Borca MV Gladue DP. Interaction of structural glycoprotein E2 of classical swine fever virus with protein phosphatase 1 catalytic subunit beta (Ppp1cb). Viruses. (2019) 11(4):307. doi: 10.3390/v11040307 30934875 PMC6521620

[16] Wang Z Nie Y Wang P Ding M Deng H. Characterization of classical swine fever virus entry by using pseudotyped viruses: E1 and E2 are sufficient to mediate viral entry. Virology. (2004) 330:332–41. doi: 10.1016/j.virol.2004.09.023 15527858

[17] Sánchez O Barrera M Farnós O Parra NC Salgado ER Saavedra PA. Effectiveness of the E2-classical swine fever virus recombinant vaccine produced and formulated within whey from genetically transformed goats. Clin Vaccine Immunol. (2014) 21:1628–34. doi: 10.1128/cvi.00416-14 PMC4248785 25274802

[18] Madera R Gong W Wang L Burakova Y Lleellish K Galliher-Beckley A. Pigs immunized with a novel E2 subunit vaccine are protected from subgenotype heterologous classical swine fever virus challenge. BMC Vet Res. (2016) 12:197. doi: 10.1186/s12917-016-0823-4 27612954 PMC5016919

[19] Lin G-J Deng M-C Chen Z-W Liu T-Y Wu C-W Cheng C-Y. Yeast expressed classical swine fever E2 subunit vaccine candidate provides complete protection against lethal challenge infection and prevents horizontal virus transmission. Vaccine. (2012) 30:2336–41. doi: 10.1016/j.vaccine.2012.01.051 22300723

[20] Lin GJ Liu TY Tseng YY Chen ZW You CC Hsuan SL. Yeast-expressed classical swine fever virus glycoprotein E2 induces a protective immune response. Vet Microbiol. (2009) 139:369–74. doi: 10.1016/j.vetmic.2009.06.027 19625145

[21] Zhou P Huang J Li Y Chen H Wu Y Fu X. Efficiency comparison of a novel E2 subunit vaccine and a classic C-strain vaccine against classical swine fever. Vet Sci. (2021) 8(8):148. doi: 10.3390/vetsci8080148 34437470 PMC8402791

[22] Hua RH Huo H Li YN Xue Y Wang XL Guo LP. Generation and efficacy evaluation of recombinant classical swine fever virus E2 glycoprotein expressed in stable transgenic mammalian cell line. PloS One. (2014) 9:e106891. doi: 10.1371/journal.pone.0106891 25198669 PMC4157854

[23] Burakova Y Madera R Wang L Buist S Lleellish K Schlup JR. Food-grade saponin extract as an emulsifier and immunostimulant in emulsion-based subunit vaccine for pigs. J Immunol Res. (2018) 2018:8979838. doi: 10.1155/2018/8979838 30599004 PMC6288570

[24] Liu ZH Xu HL Han GW Tao LN Lu Y Zheng SY. Self-assembling nanovaccine enhances protective efficacy against csfv in pigs. Front Immunol. (2021) 12:689187. doi: 10.3389/fimmu.2021.689187 34367147 PMC8334734

[25] Liu Z-H Xu H-L Han G-W Tao L-N Lu Y Zheng S-Y. A self-assembling nanoparticle: implications for the development of thermostable vaccine candidates. Int J Biol Macromol. (2021) 183:2162–73. doi: 10.1016/j.ijbiomac.2021.06.024 34102236

[26] Joshi VB Geary SM Salem AK. Biodegradable particles as vaccine delivery systems: size matters. AAPS J. (2013) 15:85–94. doi: 10.1208/s12248-012-9418-6 23054976 PMC3535111

[27] Suárez M Sordo Y Prieto Y Rodríguez MP Méndez L Rodríguez EM. A single dose of the novel chimeric subunit vaccine E2-cd154 confers early full protection against classical swine fever virus. Vaccine. (2017) 35:4437–43. doi: 10.1016/j.vaccine.2017.05.028 28688785

[28] Suárez-Pedroso M Sordo-Puga Y Sosa-Teste I Rodriguez-Molto MP Naranjo-Valdés P Sardina-González T. Novel chimeric E2cd154 subunit vaccine is safe and confers long lasting protection against classical swine fever virus. Vet Immunol Immunopathol. (2021) 234:110222. doi: 10.1016/j.vetimm.2021.110222 33690056

[29] Morales-Hernández S Ugidos-Damboriena N López-Sagaseta J. Self-assembling protein nanoparticles in the design of vaccines: 2022 update. Vaccines (Basel). (2022) 10(9):1447. doi: 10.3390/vaccines10091447 36146525 PMC9505534

[30] Chaudhary N Weissman D Whitehead KA. Mrna vaccines for infectious diseases: principles, delivery and clinical translation. Nat Rev Drug Discov. (2021) 20:817–38. doi: 10.1038/s41573-021-00283-5 PMC8386155 34433919

[31] Cai J Qiu Z Chi-Shing Cho W Liu Z Chen S Li H. Synthetic circrna therapeutics: innovations, strategies, and future horizons. MedComm (2020). (2024) 5:e720. doi: 10.1002/mco2.720 39525953 PMC11550093

[32] Niu D Wu Y Lian J. Circular rna vaccine in disease prevention and treatment. Signal Trans Target Ther. (2023) 8:341. doi: 10.1038/s41392-023-01561-x PMC10493228 37691066

[33] Zong Y Lin Y Wei T Cheng Q. Lipid nanoparticle (Lnp) enables mrna delivery for cancer therapy. Adv Mater. (2023) 35:e2303261. doi: 10.1002/adma.202303261 37196221

[34] Kiaie SH Majidi Zolbanin N Ahmadi A Bagherifar R Valizadeh H Kashanchi F. Recent advances in mrna-lnp therapeutics: immunological and pharmacological aspects. J Nanobiotech. (2022) 20:276. doi: 10.1186/s12951-022-01478-7 PMC9194786 35701851

[35] Shen L Yang J Zuo C Xu J Ma L He Q. Circular mrna-based tcr-T offers a safe and effective therapeutic strategy for treatment of cytomegalovirus infection. Mol Ther. (2024) 32:168–84. doi: 10.1016/j.ymthe.2023.11.017 PMC10787193 37974400

[36] Huang K Li N Li Y Zhu J Fan Q Yang J. Circular mrna vaccine against sars-cov-2 variants enabled by degradable lipid nanoparticles. ACS Appl Mater Interf. (2025) 17:4699–710. doi: 10.1021/acsami.4c20770 39789795

[37] Yang J Zhu J Sun J Chen Y Du Y Tan Y. Intratumoral delivered novel circular mrna encoding cytokines for immune modulation and cancer therapy. Mol Ther Nucleic Acids. (2022) 30:184–97. doi: 10.1016/j.omtn.2022.09.010 PMC9482165 36156907

[38] Huang Y-L Meyer D Postel A Tsai K-J Liu H-M Yang C-H. Identification of a common conformational epitope on the glycoprotein E2 of classical swine fever virus and border disease virus. Viruses. (2021) 13:1655. doi: 10.3390/v13081655 34452520 PMC8402670

[39] Zhu X Liu M Wu X Ma W Zhao X. Phylogenetic analysis of classical swine fever virus isolates from China. Arch Virol. (2021) 166:2255–61. doi: 10.1007/s00705-021-05084-0 34003359

[40] Qu L Yi Z Shen Y Lin L Chen F Xu Y. Circular rna vaccines against sars-cov-2 and emerging variants. Cell. (2022) 185:1728–44.e16. doi: 10.1016/j.cell.2022.03.044 35460644 PMC8971115

[41] Seephetdee C Bhukhai K Buasri N Leelukkanaveera P Lerdwattanasombat P Manopwisedjaroen S. A circular mrna vaccine prototype producing vflip-X spike confers a broad neutralization of sars-cov-2 variants by mouse sera. Antiviral Res. (2022) 204:105370. doi: 10.1016/j.antiviral.2022.105370 35772601 PMC9235288

[42] Zhou J Ye T Yang Y Li E Zhang K Wang Y. Circular Rna Vaccines against Monkeypox Virus Provide Potent Protection against Vaccinia Virus Infection in Mice. Mol Ther. (2024) 32:1779–89. doi: 10.1016/j.ymthe.2024.04.028 PMC11184329 38659224

[43] Song H Abdullah SW Pei C Shi X Chen X Ma Y. Self-assembling E2-based nanoparticles improve vaccine thermostability and protective immunity against csfv. Int J Mol Sci. (2024) 25(1):596. doi: 10.3390/ijms25010596 38203765 PMC10778992

[44] Kwak K Sohn H George R Torgbor C Manzella-Lapeira J Brzostowski J. B cell responses to membrane-presented antigens require the function of the mechanosensitive cation channel piezo1. Sci Signal. (2023) 16:eabq5096. doi: 10.1126/scisignal.abq5096 37751477 PMC10691204

[45] Scaria PV Roth N Schwendt K Muratova OV Alani N Lambert LE. Mrna vaccines expressing malaria transmission-blocking antigens pfs25 and pfs230d1 induce a functional immune response. NPJ Vaccines. (2024) 9:9. doi: 10.1038/s41541-023-00783-y 38184666 PMC10771442

[46] Curley SM Putnam D. Biological nanoparticles in vaccine development. Front Bioeng Biotechnol. (2022) 10:867119. doi: 10.3389/fbioe.2022.867119 35402394 PMC8984165

[47] López-Sagaseta J Malito E Rappuoli R Bottomley MJ. Self-assembling protein nanoparticles in the design of vaccines. Comput Struct Biotechnol J. (2016) 14:58–68. doi: 10.1016/j.csbj.2015.11.001 26862374 PMC4706605

[48] Escalona-Rayo O Zeng Y Knol RA Kock TJF Aschmann D Slütter B. *In vitro* and *in vivo* evaluation of clinically-approved ionizable cationic lipids shows divergent results between mrna transfection and vaccine efficacy. BioMed Pharmacother. (2023) 165:115065. doi: 10.1016/j.biopha.2023.115065 37406506

[49] Wang M Sun S Alberti KA Xu Q. A combinatorial library of unsaturated lipidoids for efficient intracellular gene delivery. ACS Synth Biol. (2012) 1:403–7. doi: 10.1021/sb300023h 23651337

[50] Kis Z Kontoravdi C Dey AK Shattock R Shah N. Rapid development and deployment of high-volume vaccines for pandemic response. J Adv Manuf Process. (2020) 2:e10060. doi: 10.1002/amp2.10060 33977274 PMC7361221

[51] Kim S Jeon JH Kim M Lee Y Hwang Y-H Park M. Innate Immune Responses against Mrna Vaccine Promote Cellular Immunity through Ifn-B at the Injection Site. Nat Commun. (2024) 15:7226. doi: 10.1038/s41467-024-51411-9 39191748 PMC11349762

[52] Pongma C Keawvilai P Boonmee A Wongprom B Pattarakankul T Sittplangkoon C. Effect of mrna formulated with lipid nanoparticles on the transcriptomic and epigenetic profiles of F4/80+ Liver-associated macrophages. Sci Rep. (2025) 15:1146. doi: 10.1038/s41598-025-85234-5 39774150 PMC11706949

[53] Xing J Weng L Yuan B Wang Z Jia L Jin R. Identification of a role for trim29 in the control of innate immunity in the respiratory tract. Nat Immunol. (2016) 17:1373–80. doi: 10.1038/ni.3580 PMC5558830 27695001

[54] Wang J Lu W Zhang J Du Y Fang M Zhang A. Loss of trim29 mitigates viral myocarditis by attenuating perk-driven er stress response in male mice. Nat Commun. (2024) 15:3481. doi: 10.1038/s41467-024-44745-x 38664417 PMC11045800

[55] Xing J Zhang A Du Y Fang M Minze LJ Liu YJ. Identification of poly(Adp-ribose) polymerase 9 (Parp9) as a noncanonical sensor for rna virus in dendritic cells. Nat Commun. (2021) 12:2681. doi: 10.1038/s41467-021-23003-4 33976210 PMC8113569

