# Supplementary material for: Circular mRNA-LNP vaccine encoding self-assembled E2-TMD-mi3 nanoparticles licit enhanced CSFV-specific immunity over commercial subunit vaccine
Source: Front Immunol. 2025 Jun 19;16:1604677. doi: 10.3389/fimmu.2025.1604677 (PMC12221933; doi:10.3389/fimmu.2025.1604677)
Supplement: Supplementary file 1 [file DataSheet1.docx]

Supplementary Material

## Supplementary Figures


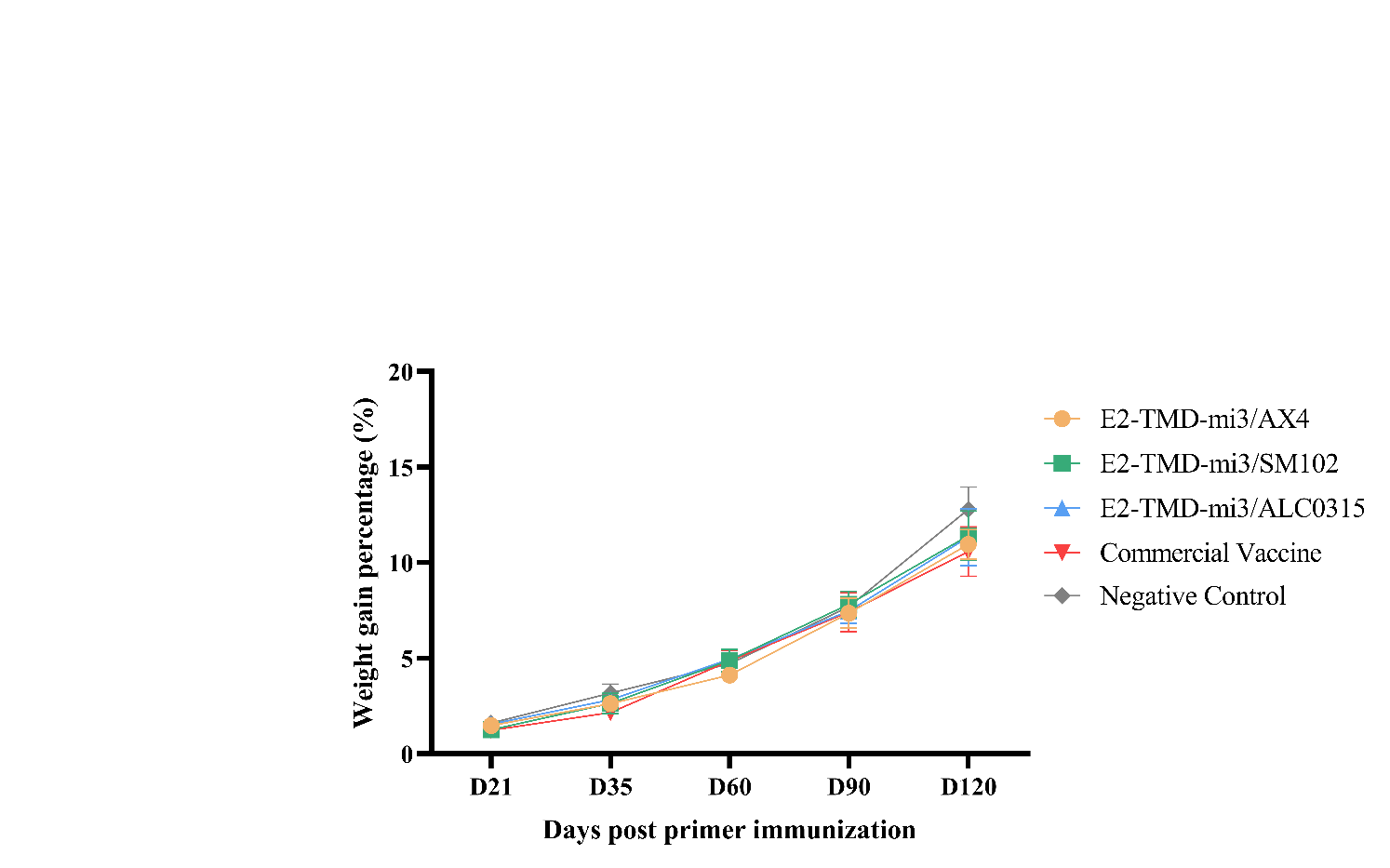


**Supplementary Figure 1.** Changes in body weight gain percentage of each cmRNA-LNP vaccine and the commercial E2 protein subunit vaccine at 21, 35, 60, 90, 120 days post-immunization (n=5 pigs per group).Statistical differences in mean antibody blocking percentages were analyzed by two-way ANOVA followed by Dunnett’s multiple comparisons test (**P* <0.05, ***P* <0.01, ****P* <0.001, *****P* <0.0001 vs. commercial E2 group). Non-significant differences (*P*≥0.05) were omitted from the figure. All data are presented as mean ±SEM (Standard Error of the Mean).
